# Androgen deprivation alone versus combined with pelvic radiation for adverse events and quality of life in clinically node-positive prostate cancer

**DOI:** 10.1038/s41598-024-54976-z

**Published:** 2024-04-08

**Authors:** Tae Hoon Lee, Hongryull Pyo, Gyu Sang Yoo, Jin Hee Kim, Seong Soo Jeon, Seong Il Seo, Byong Chang Jeong, Hwang Gyun Jeon, Hyun Hwan Sung, Minyong Kang, Wan Song, Jae Hoon Chung, Won Park

**Affiliations:** 1 Department of Radiation Oncology, Samsung Medical Center, Sungkyunkwan University School of Medicine, 81 Irwon-ro, Gangnam-gu, Seoul, 06351 Republic of Korea; 2 Department of Radiation Oncology, Chungbuk National University Hospital, Cheongju, Republic of Korea; 3 Department of Radiation Oncology, Dongsan Medical Center, Keimyung University School of Medicine, Daegu, Republic of Korea; 4 Department of Urology, Samsung Medical Center, Sungkyunkwan University School of Medicine, Seoul, Republic of Korea

**Keywords:** Prostate cancer, Prostate cancer

## Abstract

The COHORT trial was conducted to compare the efficacy of androgen deprivation therapy (ADT) alone versus combined with radiation therapy (ADT + RT) for clinically node-positive prostate cancer. We reported adverse events and quality of life between the two treatment groups. Fifty-nine patients were randomized to receive ADT alone or ADT + RT and analyzed as per-protocol. Patients allocated to the ADT alone arm received ADT for at least 2 years. Patients in the ADT + RT arm received additional pelvic RT. Higher rates of grade ≥ 2 acute genitourinary (0% vs. 7.1%) and late gastrointestinal adverse events (0% vs. 14.3%) were reported in the ADT + RT arm compared with the ADT alone. However, grade ≥ 2 late genitourinary toxicity was more common in the ADT alone than the ADT + RT arm (9.7% vs. 3.6%). No grade ≥ 3 adverse events were reported. There was no statistically significant difference in EPIC scores between two treatment arms. However, the urinary and bowel domains tended to decrease and recover in the ADT + RT arm. In conclusion, ADT + RT demonstrated higher rates of adverse events compared to ADT alone. However, the addition of RT did not significantly impact the quality of life.

## Introduction

Prostate cancer is the second most frequently diagnosed cancer in men worldwide after lung cancer^1^. Although most cases of prostate cancer are diagnosed while localized, approximately 10% of patients present with regional lymph node metastasis^2^. Prostate cancer is one of the most common cancers treated by radiation therapy (RT)^3^. The role of RT in organ-confined high-risk prostate cancer is well-established^4,5^. However, the role of RT in node-positive prostate cancer is controversial, as high-level evidence is lacking. Retrospective population studies comparing local therapy, including RT, versus no local therapy have demonstrated a survival benefit of local therapy^6,7^. An analysis of data from the control arm of the STAMPEDE trial reported a failure-free survival benefit of RT in the N + M0 subcohort^8^. Although RT may be the preferred treatment option for clinically node-positive prostate cancer based on these retrospective analyses, confirmatory randomized evidence is required. To address this question, we designed the COHORT trial, a multi-institutional randomized phase III trial aimed at determining the potential benefits of ADT plus RT (ADT + RT) for clinically pelvic lymph node-positive prostate cancer.

Although the efficacy of the treatment modalities is the most important, adverse events and their impact on the quality of life cannot be disregarded, as the risks and benefits of the treatment need to be thoroughly considered in clinical settings. In previous randomized trials, adding RT to ADT increased treatment-related adverse events, especially urinary and gastrointestinal symptoms^4,9^. For node-positive prostate cancer, the RT field often extends to the pelvic regional lymph node area, and the negative impact of RT may be more severe. Therefore, adverse events of RT and their impact on the quality of life in this subgroup of patients need to be addressed. The purpose of this analysis is to compare genitourinary and gastrointestinal adverse events and the quality of life of patients with node-positive prostate cancer who underwent ADT alone and those who underwent ADT + RT.

## Materials and methods

### Patients

The inclusion criteria for this trial were as follows: pathological confirmation of prostate cancer within 6 months before enrollment; radiological evidence (computed tomography [CT] and magnetic resonance imaging [MRI]) of suspected metastatic pelvic lymph node (short axis ≥ 0.5 cm) that decreased in size after 2–3 months of ADT, indicating at least partial response according to Response Evaluation Criteria in Solid Tumors version 1.1^10^; age ≥ 20 years; Eastern Cooperative Oncology Group performance status of 0–1; satisfactory complete blood count test result within 6 months (absolute neutrophil count ≥ 1500 cells/mm^3^, platelets ≥ 50,000 cells/mm^3^, hemoglobin ≥ 8.0 g/dL); satisfactory renal function test within 6 months (creatinine < 2.0 ng/dL); and satisfactory liver function test within 6 months (total bilirubin < 1.5 times the upper reference value of the participating institution; alanine aminotransferase or aspartate aminotransferase < 2.5 times the upper reference value of the participating institution).

The exclusion criteria were as follows: distant metastasis; receipt of hormone therapy more than 6 months before enrollment; history of other definitive treatments for prostate cancer, such as radical prostatectomy; history of prior pelvic RT; and history of cancer other than skin or thyroid cancer. The accrual was conducted from July 2016 until July 2021.

### Study design and treatments

The trial was registered at ClinicalTrials.gov on 07/08/2017 (NCT03241537). Patients were screened for enrollment by treating radiation oncologists when they received the first ADT. If the lymph node size reduction was confirmed to be more than partial response after 2–3 months of ADT, patients were enrolled in the trial and were randomly assigned to either the ADT alone arm or the ADT + RT arm in a 1:1 ratio. The block randomization method was used to prepare a randomization table. Patients in the ADT alone arm continued to receive ADT alone, whereas those in the ADT + RT arm underwent both ADT and pelvic external beam RT. ADT was administered using a combination of a gonadotropin-releasing hormone (GnRH) agonist and anti-androgen. At least 2 years of ADT was required unless it was deemed intolerable by the treating clinicians due to adverse events. The extended use of ADT was allowed.

Patients allocated to the ADT + RT arm underwent simulation CT for RT planning. Intensity-modulated RT is required for RT planning. The high-risk clinical target volume (HR-CTV) included the entire prostate, the involved extraprostatic tissue, seminal vesicles, and metastatic pelvic lymph nodes. For cT3a disease, proximal seminal vesicles were included in the HR-CTV. For cT3b and cT4 disease, the entire seminal vesicles were included in the HR-CTV. The low-risk (LR) CTV included the obturator, external iliac, internal iliac, and presacral lymphatic areas according to Radiation Therapy Oncology Group (RTOG) consensus^11^ published in 2009, which was available at the time of protocol establishment. The CTV-to-planning target volume (PTV) expansion was 3–5 mm for the prostate and seminal vesicle region and 5–7 mm for the nodal area. The prescribed dose to the HR-PTV was no less than the biologically effective dose (BED) of 170 Gy with an alpha–beta ratio of 1.5, except for the pelvic metastatic lymph nodes. A BED of no less than 120 Gy was required for the lymph nodes. The dose prescribed for the LR-PTV was not less than 100 Gy. Institutional dose fractionation regimens were permitted if the study requirements were met. In practice, most of the patients received 70 Gy to the prostate with or without seminal vesicles, 50.4 Gy to the pelvic nodal basin, and 59.92–61.6 Gy to suspected lymph nodes in 28 fractions. The plan was optimized to cover 95% of the PTV with 100% of the prescribed dose. Organs-at-risk (OARs) included the bladder, rectum, and penile bulb and were delineated according to RTOG guidelines^12^. The dose constraints for the OARs are summarized in Supplementary Table 1. RT was initiated within 10 days of the simulation CT scans. The fractions were delivered 5 days per week. RT should be completed within 10 weeks of its initiation. Pelvic RT was permitted as a salvage treatment in the ADT alone arm if needed.

The patients were followed up at 6-months intervals for 3 years from enrollment and once a year thereafter. The required follow-up period for oncologic outcomes was 5 years. Adverse event evaluations, and quality of life assessments were performed at every follow-up visit.

### Endpoints and statistical analysis

Genitourinary and gastrointestinal adverse events were classified as acute (occurring within < 6 months) or late (occurring ≥ 6 months) based on the onset from the start of the treatment and graded according to Common Terminology Criteria for Adverse Events version 4.0. Quality of life was assessed using the Expanded Prostate Index Composite (EPIC)^13^. The rates of acute and late gastrointestinal and genitourinary adverse events were compared between the two arms using the chi-squared test. The EPIC was scored for each domain (urinary, bowel, sexual, and hormonal) and subscale. Changes in each domain and subscale score from enrollment were compared using a linear mixed model with the allocated arm and time as fixed effects and each patient as a random effect. The scores for each domain and subscale at 12, 24, and 36 months were compared with the baseline scores measured at enrollment using the Wilcoxon rank sum test. These analyses were performed as per-protocol.

The sample size for this trial was calculated based on the assumption that the 5-years recurrence-free survival rate would be 40% in the ADT alone arm and 80% in the ADT + RT arm. Sixty participants were required, using a log-rank test with a two-sided significance level of 5%, 80% statistical power, and a 10% dropout rate. Statistical significance was defined as a *p*-value < 0.05. All statistical analyses were conducted using R software (version 4.2.1; The R Foundation for Statistical Computing, Vienna, Austria).

### Ethics approval and consent to participate

This study was approved by the Institutional Review Board of Samsung Medical Center (no. 2015-11-139) before trial initiation. Written informed consent was obtained from each participant before enrollment. All procedures performed in this study were in accordance with the ethical standards of the institutional and national regulations and with the 1964 Helsinki Declaration and its later amendments.

## Results

### Patient characteristics and treatment

**Figure 1:**
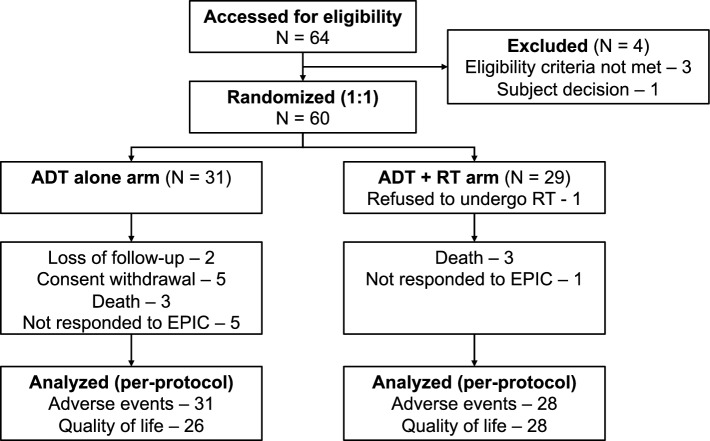
CONSORT diagram. ADT, androgen deprivation therapy; EPIC, Expanded Prostate Index Composite; RT, radiation therapy.

This study enrolled 60 patients: 31 allocated to the ADT alone arm and 29 allocated to the ADT + RT arm. One patient in the ADT + RT arm refused to undergo RT and was consequently excluded from the per-protocol analysis, leaving 59 patients in the analysis. A CONSORT diagram is shown in Fig. 1. The median follow-up duration was 3.31 years (range: 0.24–6.87 years). Seven (22.6%) patients in the ADT alone arm were either lost to follow-up (N = 2) or withdrew their consent (N = 5), while no patients in the ADT + RT arm were removed from the follow-up. However, no statistical difference in follow-up duration was reported (ADT alone arm: median 2.99 years; ADT + RT arm: median 3.57 years; *p* = 0.106). The baseline characteristics of the patients and their treatment specifics are summarized in Table 1. The patient characteristics were well balanced between the two arms. The median age of all patients was 70 years (range: 49–83 years). The median initial PSA was 35.9 ng/mL (range: 3.2–1032 ng/mL). Most patients (84.7%) had a Gleason score of ≥ 8, and all patients had T3 or T4 disease. The median number of metastatic pelvic lymph nodes was two (range: 1–7), and the median lymph node size was 12 mm (range: 5–41 mm).

**Table 1:** Patient characteristics and treatment.

Characteristics	ADT alone (N = 31)	ADT + RT (N = 28)	*P*-value
Age (median, years)	71 (range, 61–79)	69.5 (range, 49–83)	0.722
ECOG performance status			0.397
0	19 (61.3%)	21 (75.0%)	
1	12 (38.7%)	7 (25.0%)	
Initial PSA (median, ng/mL)	38.3 (range, 5.0–1032)	35.5 (range, 3.2–370.2)	0.369
Gleason score			0.438
6	0 (0.0%)	1 (3.6%)	
7	5 (16.1%)	3 (10.7%)	
8	11 (35.5%)	15 (53.6%)	
9	12 (38.7%)	8 (28.6%)	
10	3 (9.7%)	1 (3.6%)	
T stage			0.212
T3a	6 (19.4%)	7 (25.0%)	
T3b	18 (58.1%)	10 (35.7%)	
T4	7 (22.6%)	11 (39.3%)	
Number of lymph node metastasis (median)	2 (range, 1–7)	2 (range, 1–5)	0.436
Maximum size of metastatic lymph node (median, mm)	12.4 (range. 6.0–41.0)	11.9 (range, 5.0–25.1)	0.471
ADT medication			0.515
Leuprolide + bicalutamide	20 (64.5%)	15 (53.6%)	
Triptorelin + bicalutamide	9 (29.0%)	12 (42.9%)	
Goserelin + bicalutamide	2 (6.5%)	1 (3.6%)	
ADT duration (median, years)	2.99 (range, 0.24–6.58)	2.65 (range, 1.55–5.75)	0.502
ADT cessation during follow-up	0 (0.0%)	6 (21.4%)	0.022
RT total dose			–
77 Gy	–	1 (3.6%)	
70 Gy	–	26 (92.9%)	
67.2 Gy	–	1 (3.6%)	
RT fractionation			–
28	–	27 (96.4%)	
33	–	1 (3.6%)	

ADT, androgen deprivation therapy; ECOG, Eastern Cooperative Oncology Group; PSA, prostate-specific antigen; RT, radiation therapy.

Leuprolide (59.3%), triptorelin (35.6%), and goserelin (5.1%) were initially used as the GnRH agonists in ADT. All patients were administered bicalutamide as an anti-androgen. The median duration of ADT for all patients was 2.69 years (range: 0.24–6.58 years). A total of six (10.2%) stopped taking the ADT during follow-up. All six patients who stopped taking ADT were in the ADT + RT arm. Among them, five stopped ADT after 2 years at the discretion of the clinicians. One patient discontinued ADT because of a suspected drug rash. For RT, 26 of 28 patients underwent treatment with a dose fractionation of 70 Gy in 28 fractions. One patient received 67.2 Gy in 28 fractions due to a high rectal dose, and another patient received 77 Gy in 33 fractions.

### Adverse events

The acute and late gastrointestinal and genitourinary adverse events are summarized in Table 2. All reported gastrointestinal and genitourinary adverse events were grade 1 or 2. There was no grade ≥ 3 in acute and late adverse events. A statistically significant difference in the rates of acute genitourinary and late gastrointestinal adverse events between the two treatment arms were reported (*p* < 0.001). The incidence of acute grade 2 genitourinary adverse events was 0% in the ADT alone and and 7.1% in the ADT + RT arm. For the late grade 2 gastrointestinal adverse events, the rates were 0% in the ADT alone arm and 14.3% in the ADT + RT arm.

**Table 2:** Highest grades of gastrointestinal and genitourinary adverse events.

Adverse events	ADT alone (N = 31)	ADT + RT (N = 28)	*P*-value
Acute gastrointestinal, highest grade			0.143
0	27 (87.1%)	20 (67.9%)	
1	4 (12.0%)	9 (32.1%)	
Acute genitourinary, highest grade			< 0.001
0	22 (71.0%)	1 (3.6%)	
1	9 (29.0%)	25 (89.3%)	
2	0 (0.0%)	2 (7.1%)	
Late gastrointestinal, highest grade			< 0.001
0	27 (87.1%)	8 (28.6%)	
1	4 (12.9%)	16 (57.1%)	
2	0 (0.0%)	4 (14.3%)	
Late genitourinary, highest grade			0.084
0	19 (61.3%)	11 (39.3%)	
1	9 (29.0%)	16 (57.1%)	
2	3 (9.7%)	1 (3.6%)	

ADT, androgen deprivation therapy; RT, radiation therapy.

For acute genitourinary toxicities, two patients reported grade 2 adverse events. Both patients experienced frequency, and one patient experienced urgency. These symptoms spontaneously subsided to grade 1, and at the 18-months visit, both patients no longer reported any genitourinary adverse events. Regarding late gastrointestinal toxicities, a total of 4 patients reported grade 2 adverse events. Three patients experienced moderate rectal hemorrhage; however, only one patient required elective argon plasma coagulation, and the rectal hemorrhage regressed to intermittent grade 1 events for all three patients. Two patients reported proctitis, and one patient reported dyspepsia. The proctitis and dyspepsia events were temporary. As for late genitourinary toxicities, four patients reported grade 2 adverse events. The symptoms included frequency, urgency, pain, incontinence, and obstructive symptoms. However, these symptoms were not persistent.

### Quality of life

An analysis of the quality of life based on EPIC scores was conducted in 54 patients. All 59 patients responded to the EPIC during enrollment, but five declined to respond to the EPIC during follow-up. Additionally, two patients refused to respond to the sexual domain questions during enrollment. The median baseline EPIC scores for the urinary, bowel, sexual, and hormonal domains during enrollment were 87.5 (range: 52.1–100.0), 98.2 (range: 75.0–100.0), 27.5 (range: 1.9–56.4), and 88.6 (range: 45.5–100.0), respectively. The baseline EPIC scores for each domain, as well as each subscale, did not show statistically significant differences between the two groups.

**Figure 2:**
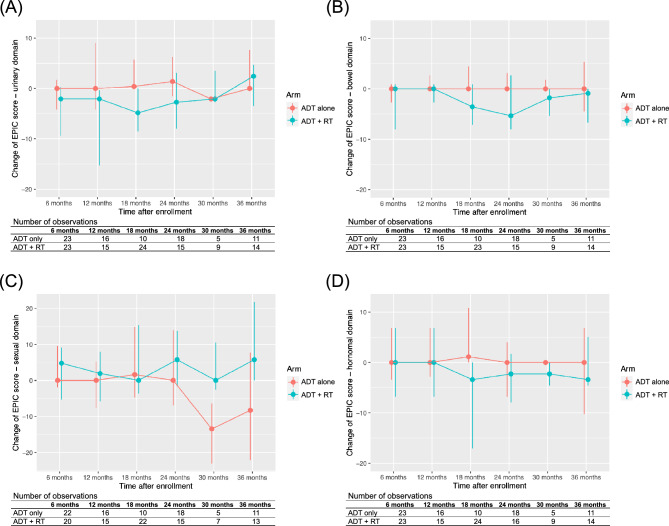
Change of Expanded Prostate Index Composite score from enrollment for (**A**) urinary domain, (**B**) bowel domain, (**C**) sexual domain, and (**D**) hormonal domain. Dots represent median values, while vertical lines represent interquartile ranges. ADT, androgen deprivation therapy; RT, radiation therapy.

The change in the EPIC score from enrollment in each domain is shown in Fig. 2. As not all patients responded to the EPIC during follow-up, the number of observations is shown in each panel of the figure. In the linear mixed model analysis, no statistically significant association was observed between the allocated arms and the EPIC score for each domain (urinary, *p* = 0.202; bowel, *p* = 0.050; sexual, *p* = 0.270; hormonal, *p* = 0.069). When analyzed with the 10 subscales, all subscales did not show a statistically significant difference between the two treatment arms (Supplementary Fig. 1).

Domain and subscale scores at 12, 24, and 36 months for both treatment arms are summarized in Table 3. When compared with the baseline scores, the scores at 12, 24, and 36 months in the ADT alone arm did not show a statistically significant. In the ADT + RT arm, the urinary function (*p* = 0.013) and incontinence (*p* = 0.004) subscales were significantly lower than the baseline at 12 months, and the bowel bother subscale was significantly lower at 24 months (*p* = 0.031). However, these scores did not show a significant difference from the baseline at 36 months. On the other hand, the overall sexual domain score (*p* = 0.026) and sexual bother subscale (*p* = 0.004) were significantly higher than the baseline at 36 months.

**Table 3:** Expanded Prostate Index Composite score for each domain and subscale by study arm and time.

Domain and subscale	ADT alone (N = 31)							ADT + RT (N = 28)						
	Baseline	12 months		24 months		36 months		Baseline	12 months		24 months		36 months	
	Median (range)	Median (range)	*P*-value*	Median (range)	*P*-value*	Median (range)	*P*-value*	Median (range)	Median (range)	*P*-value*	Median (range)	*P*-value*	Median (range)	*P*-value*
Urinary	86.8 (57.7–100.0)	90.6 (50.7–100.0)	0.623	89.3 (57.7–100.0)	0.600	95.8 (27.8–100.0)	0.620	88.2 (52.1–100.0)	84.8 (67.3–97.9)	0.070	89.6 (74.3–100.0)	0.603	91.7 (63.9–100.0)	0.393
Function	93.4 (66.6–100.0)	100.0 (51.6–100.0)	0.351	95.0 (66.6–100.0)	0.390	95.0 (26.6–100.0)	0.516	95.0 (70.0–100.0)	86.6 (53.4–100.0)	**0.013**	93.4 (71.6–100.0)	0.140	100.0 (78.4–100.0)	0.308
Bother	82.1 (46.4–100.0)	85.7 (50.0–100.0)	0.733	85.7 (46.4–100.0)	0.811	96.4 (28.6–100.0)	0.690	85.7 (39.3–100.0)	85.7 (60.7–96.4)	0.391	85.7 (67.9–100.0)	0.706	85.7 (53.6–100.0)	0.709
Incontinence	91.8 (39.5–100.0)	100.0 (45.8–100.0)	0.366	96.9 (45.8–100.0)	0.840	91.8 (8.3–100.0)	0.973	96.9 (64.5–100.0)	83.3 (58.5–100.0)	**0.004**	91.8 (60.5–100.0)	0.139	100.0 (66.8–100.0)	0.959
Irritative/obstructive	85.7 (46.4–100.0)	89.3 (57.1–100.0)	0.820	91.1 (64.3–100.0)	0.655	96.4 (35.7–100.0)	0.664	89.3 (32.1–100.0)	85.7 (71.4–100.0)	0.645	92.9 (75.0–100.0)	0.627	91.1 (67.9–100.0)	0.339
Bowel	96.4 (75.0–100.0)	98.2 (94.6–100.0)	0.299	98.2 (89.3–100.0)	0.451	94.6 (62.5–100.0)	0.892	98.2 (87.5–100.0)	94.6 (62.5–100.0)	0.484	94.6 (83.9–100.0)	0.100	92.9 (69.6–100.0)	0.202
Function	96.4 (57.1–100.0)	100.0 (92.9–100.0)	0.078	96.4 (85.7–100.0)	0.227	96.4 (64.3–100.0)	0.396	98.2 (82.1–100.0)	92.9 (78.6–100.0)	0.558	91.1 (85.7–100.0)	0.440	89.3 (67.9–100.0)	0.197
Bother	100.0 (64.3–100.0)	100.0 (89.3–100.0)	0.271	100.0 (82.1–100.0)	0.897	100.0 (60.7–100.0)	0.541	100.0 (82.1–100.0)	96.4 (39.3–100.0)	0.367	92.9 (78.6–100.0)	**0.031**	98.2 (60.7–100.0)	0.126
Sexual	28.8 (1.9–56.4)	30.8 (1.9–64.8)	0.636	29.8 (1.9–51.3)	0.703	26.9 (6.4–31.4)	0.263	25.0(1.9–50.0)	30.8 (6.4–44.8)	0.721	30.8 (1.9–41.0)	0.297	30.8 (25.0–48.1)	**0.026**
Function	2.8 (0.0–43.6)	0.0 (0.0–57.4)	0.555	0.0 (0.0–51.9)	0.052	2.8 (0.0–14.8)	0.281	0.0 (0.0–61.1)	3.7 (0.0–39.8)	0.793	2.8 (0.0–31.4)	0.825	2.8 (0.0–25.9)	0.958
Bother	56.3 (6.3–100.0)	75.0 (6.3–100.0)	0.924	81.3 (6.3–100.0)	0.261	81.3 (12.5–100.0)	0.716	53.1 (6.3–100.0)	81.3 (6.3–100.0)	0.430	68.8 (6.3–100.0)	0.112	93.8 (43.8–100.0)	**0.004**
Hormonal	88.6 (45.5–100.0)	93.2 (68.2–100.0)	0.924	93.2 (54.5–100.0)	1.000	88.6 (54.5–100.0)	0.921	90.9 (54.5–100.0)	84.1 (59.1–100.0)	0.647	88.6 (54.5–100.0)	0.496	92.0 (72.7–100.0)	0.659
Function	80.0 (40.0–100.0)	85.0 (65.0–100.0)	0.894	87.5 (50.0–100.0)	0.686	80.0 (40.0–100.0)	0.921	85.0 (40.0–100.0)	80.0 (55.0–100.0)	0.851	87.5 (50.0–100.0)	0.635	90.0 (75.0–100.0)	0.870
Bother	91.7 (41.7–100.0)	100.0 (70.8–100.0)	0.380	95.8 (58.3–100.0)	0.506	91.7 (50.0–100.0)	0.567	93.8 (62.5–100.0)	87.5 (62.5–100.0)	0.224	91.7 (50.0–100.0)	0.655	91.7 (70.8–100.0)	0.715

*Calculated between the baseline scores and the scores at the specified time.

ADT, androgen deprivation therapy; RT, radiation therapy.

*P*-values lower than 0.05 were marked in bold.

## Discussion

The COHORT trial was designed to evaluate the differences in treatment outcomes, adverse events, and quality of life between ADT alone and ADT + RT. Currently available clinical guidelines prefer definitive RT with ADT in node-positive prostate cancer^14–16^. However, no randomized evidence was available for patients with node-positive prostate cancer, and reports of adverse events and quality of life in these patients were scarce. In this analysis, statistically higher crude rates of acute genitourinary and late gastrointestinal adverse events in the ADT + RT arm were shown. However, most of the reported adverse events were mild, and there were no grade ≥ 3 events. Furthermore, the impact of adding pelvic RT on the quality of life appears to be temporary and limited. This report adds valuable reference for evaluating risk and benefit when making clinical decisions for node-positive prostate cancer, as the patient cohort in this study had well-balanced characteristics between the two arms, and patients in the same arm were generally treated homogeneously.

The crude rates of adverse events were higher in the ADT + RT arm in this trial, as expected based on previous trials comparing ADT alone versus ADT + RT in high-risk localized prostate cancer settings^4,9^. Crude rates only show the number of patients who experienced certain adverse events during the follow-up period, without considering recovery. In this trial, changes in the EPIC score for each domain from enrollment were analyzed, which may show the temporal trend of the functional changes evaluated by the patient. The changes in the urinary and bowel domains of the EPIC score were not significantly different between the two arms. Although not statistically significant, the urinary domain of the EPIC was lower in the ADT + RT arm until 24 months of treatment, with a similar trend in the bowel domain at the 18- and 24-months evaluations. However, these scores recovered afterward. Similar trends were observed in the temporal analysis of the subscales. At 12 months, the function and incontinence subscales of the urinary domain were lower than the baseline, and the bother subscale of the bowel domain was lower than the baseline in the ADT + RT arm. However, these subscales at 36 months were not different from the baseline, suggesting possible recovery. Although adding RT to ADT can increase the occurrence of adverse events, these effects may not persist in the long term, and the impact on the quality of life would be limited. Previous randomized trials that investigated adding pelvic RT in various clinical settings also reported that although pelvic RT was associated with more adverse events, the effect of adding pelvic RT on the quality of life was limited^17–19^, and these results are in concordance with the results of this analysis. Clinicians may not have to hesitate to apply RT to clinically node-positive prostate cancer due to concerns about genitourinary and gastrointestinal toxicities.

The baseline sexual domain of the EPIC was low in both arms, with a median of 27.5 out of 100. Although not statistically significant, the sexual domain in the ADT alone arm decreased further in 30–36 months, which may be associated with the extended use of ADT in this arm. The 36-months sexual domain score and sexual bother subscale were significantly higher than the baseline in the ADT + RT arm, which may be attributed to a higher rate of ADT discontinuation in this arm. Although the role of ADT prolongation for > 3 years in definitive treatment settings has not yet been established and remains under debate^20,21^, some clinicians in this trial preferred extending ADT, especially when other definitive treatment modalities were not applied. Both ADT and RT can have a detrimental effect on sexual function^22,23^. Based on the observations of this trial, continuous usage of ADT may have a more significant impact on decreasing sexual function than the long-term effects of RT. However, only a small number of patients were available for long-term sexual function evaluation in this trial, and more patients would be needed to obtain confirmative results.

This study has several limitations that must be acknowledged. Firstly, protocol adherence in the ADT alone arm was low, with seven patients withdrawing or being lost to follow-up. The follow-up required visits to the radiation oncology department for questionnaire completion, but some patients who were only followed up clinically at the urology department did not comply with the protocol and chose to withdraw. This may have led to the underreporting of late adverse events in the ADT alone arm. Secondly, the duration of ADT was not strictly determined in the trial protocol, and the rate of ADT cessation during follow-up differed between the two arms. The protocol specified a minimum ADT duration of 2 years, and the decisions of treating clinicians regarding the ADT duration could differ, given that patients in the two groups did not undergo the same treatment. These varying patterns of ADT usage may have influenced adverse events and quality of life outcomes. Finally, patient adherence to the EPIC questionnaire request was low, even though it was planned for every patient at every follow-up visit. This was often due to patients refusing to visit the radiation oncology department for the questionnaire while attending the urology department for clinical follow-up. Limited adherence to the EPIC questionnaire can hinder the representativeness of the quality of life results in this trial. Despite these limitations, this trial provides new high-level evidence for the treatment of patients with clinically node-positive prostate cancer.

In conclusion, the current analysis of the COHORT trial has reported a higher incidence of acute genitourinary and late gastrointestinal adverse events in the ADT + RT arm compared to the ADT alone arm. Nonetheless, no severe adverse events were reported, and the grade 2 adverse events that did occur were not persistent. In the quality of life analysis using the EPIC, the deterioration of urinary and bowel domain scores eventually started to recover after 2 years from the treatment. We look forward to the maturation of the COHORT trial for the long-term clinical outcomes.

### Supplementary Information

Supplementary Information.

## Data Availability

The datasets generated and/or analysed during the current study are not publicly available due the potential disclosure of personal information of the participants but are available from the corresponding author on reasonable request.
