# Supplementary material for: Androgen deprivation alone versus combined with pelvic radiation for adverse events and quality of life in clinically node-positive prostate cancer
Source: Sci Rep. 2024 Apr 8;14:8207. doi: 10.1038/s41598-024-54976-z (PMC11001889; doi:10.1038/s41598-024-54976-z)
Supplement: Supplementary file 1 — Supplementary Information. [file 41598_2024_54976_MOESM1_ESM.pdf]

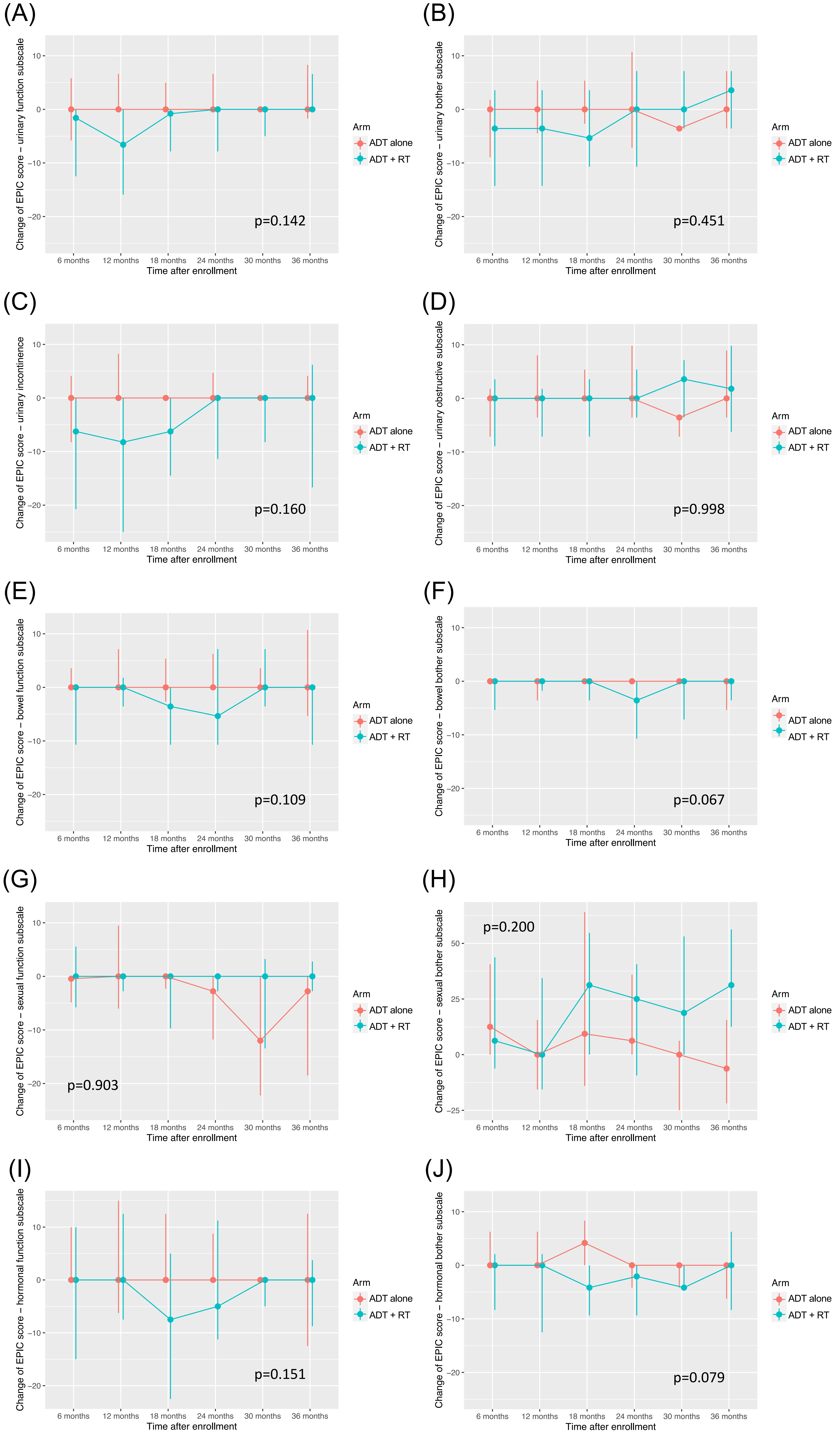

Supplementary Figure 1. Change of ten subscale of Expanded Prostate Index Composite (EPIC) score from enrollment. (A) Urinary function, (B) urinary bother, (C) urinary incontinence, (D) urinary irritative/obstructive, (E) bowel function, (F) bowel bother, (G) sexual function, (H) sexual bother, (I) hormonal function, and (J) hormonal bother subscales.

Dots represent median values, while vertical lines represent interquartile ranges. The p-values represent the intergroup difference between the two treatment groups and were calculated based on a linear mixed model. ADT, androgen deprivation therapy; RT, radiation therapy

Supplementary Table 1. Radiation dose constraints to organs-at-risk

Normal organ	Dose to < 15% volume	Dose to < 25% volume	Dose to < 35% volume	Dose to < 50% volume
Bladder (BED)	126 Gy	117 Gy	107 Gy	97 Gy
Rectum (BED)	117 Gy	107 Gy	97 Gy	87 Gy
Penile bulb (BED)	Mean dose less than or equal to 73.0 Gy			

BED was calculated with alpha-beta ratio of 3.

Abbreviation: BED, biologically effective dose.
